# Integrating Habitat Suitability and Soil Heavy-Metal Contamination to Identify Conservation Priorities for the Mongolian Wild Ass

**DOI:** 10.3390/biology15211960

**Published:** 2026-11-01

**Authors:** Bingqi Dang, Abdugheni Abliz, Jinting Liu, Xinyu Shi, Xu Li, Jialin Li

**Affiliations:** 1 College of Geography and Remote sensing Sciences, Xinjiang University, Urumqi 830046, China; 107552403528@stu.xju.edu.cn (B.D.); liujinting@stu.xju.edu.cn (J.L.); shixinyu@stu.xju.edu.cn (X.S.); 107552401129@stu.xju.edu.cn (X.L.); lijialin@stu.xju.edu.cn (J.L.); 2 Xinjiang Key Laboratory of Oasis Ecology, Xinjiang University, Urumqi 830046, China

**Keywords:** arid region, wildlife conservation, Mongolian wild ass, habitat suitability, climate change, heavy-metal pollution, species distribution model

## Abstract

In arid regions, water scarcity and limited vegetation regeneration capacity make the habitats of large herbivores particularly vulnerable to climate change and human activities. However, conventional habitat-suitability assessments may overlook potential environmental pressures associated with soil heavy-metal contamination. This study used the BIOMOD2 ensemble modeling framework to predict potential habitat suitability for the Mongolian wild ass under current environmental conditions and future climate scenarios. Current habitat-suitability predictions were further overlaid with soil heavy-metal contamination classes derived from the Nemerow integrated pollution index to examine their spatial correspondence. Under four-fold spatial block cross-validation, the EMca ensemble achieved an AUC of 0.893 ± 0.032 and a TSS of 0.610 ± 0.048. Distance to water sources was the most influential predictor, accounting for 43.76% of the total normalized variable importance. Highly suitable habitat was projected to decline from 0.4637 × 10^4^ km^2^ under current conditions to 0–0.1180 × 10^4^ km^2^ across future climate scenarios, with particularly pronounced reductions under SSP370 and SSP585 during the middle and late future periods. The spatial overlay analysis showed that 73.56% of current highly suitable habitat overlapped with light pollution, 24.31% with warning-level contamination, and 2.12% with moderate pollution. These findings indicate that high habitat suitability does not necessarily correspond to low soil-contamination levels. By integrating habitat suitability and soil-contamination patterns, this study provides a spatial basis for identifying conservation-priority habitats and areas requiring targeted environmental monitoring in Kalamaili National Park.

## Simple Summary

The Mongolian wild ass is an important large herbivore in Kalamaili National Park, an arid region of northwestern China. Its habitat is increasingly affected by climate change and human activities, while soil heavy-metal contamination may pose an additional environmental concern. In this study, we predicted habitat suitability for the Mongolian wild ass under current conditions and future climate scenarios and separately examined the spatial overlap between current habitat suitability and soil heavy-metal contamination. Distance to water sources was the most influential predictor of habitat suitability. Under future climate scenarios, areas of highly suitable habitat were projected to decrease, while low-suitability habitat accounted for a larger proportion of the remaining suitable area. Under current conditions, a small proportion of highly suitable habitat overlapped with areas of moderate soil contamination. These findings indicate that high habitat suitability does not necessarily correspond to the lowest soil-contamination levels. Considering both habitat suitability and soil-contamination conditions can therefore help identify conservation-priority habitats and areas where pollution monitoring and environmental management should be strengthened.

## 1. Introduction

Ongoing changes in the global climate, together with the continued expansion of human-induced disturbances, have placed growing pressure on biodiversity conservation [1,2]. Habitat suitability and species distributions have been significantly affected by increasingly frequent extreme weather events, changes in precipitation patterns, and rising temperatures [1,3]. Meanwhile, escalating anthropogenic influences, including mineral exploitation, transportation, and industrial production, have contributed to heavy-metal contamination of soils and water bodies through waste residue accumulation, atmospheric deposition, and traffic-related dust [4,5,6]. Owing to their environmental persistence, non-degradability, and bioaccumulative characteristics, the long-term accumulation of heavy metals has become an important factor restricting ecosystem health [7]. These pressures may be particularly pronounced in arid and semi-arid ecosystems, where water scarcity and relatively low vegetation productivity make ecological structure and functioning highly sensitive to environmental change. Climate change may further affect water availability and vegetation productivity, while heavy-metal contamination may introduce additional environmental pressures on wildlife habitats [8,9,10].

In China, the Mongolian wild ass (*Equus hemionus*) is listed as a first-class nationally protected wild animal [11,12]. Historical assessments indicated that the species was once widely distributed across northwestern China, although its distribution range and population size had already undergone substantial declines [13]. Kalamaili National Park is an important distribution area and core habitat for the Mongolian wild ass in China, and protecting habitats in this region is essential for maintaining the stability of its population. In recent years, climate change, combined with human activities such as resource extraction and the construction of transportation infrastructure, has modified the regional ecological environment and may result in the contraction, displacement, or fragmentation of suitable habitats for the Mongolian wild ass [14]. Previous research in Kalamaili National Park has documented heavy-metal contamination in soils and substantial Cr accumulation in several dominant plant species [15]. For grazing herbivores, contaminated forage and the incidental ingestion of soil during feeding are potential pathways of heavy-metal exposure [16]. Given evidence that such exposure can adversely affect livestock health and reproductive performance [17], soil contamination warrants consideration when assessing habitat conditions for the Mongolian wild ass.

Identifying potentially suitable habitats is essential for understanding habitat distribution and developing effective conservation strategies. Commonly used species distribution modeling (SDM) algorithms include GLM, GAM, GBM, RF, and MaxEnt, among others, and different algorithms vary in their ability to characterize species–environment relationships [18,19]. Compared with single models, ensemble models can integrate the results of multiple algorithms and may reduce model uncertainty and improve prediction stability; therefore, they have been widely used in species distribution modeling and climate change impact assessments [20,21]. Previous studies on habitat suitability for the Mongolian wild ass have primarily focused on environmental factors such as climate, water availability, topography, vegetation, and human activities [14,22,23]. However, the spatial correspondence between predicted suitable habitats and soil heavy-metal contamination in Kalamaili National Park remains less well-understood. Consequently, habitat-suitability assessments alone may not fully reflect the soil-contamination conditions within potentially suitable habitats.

On this basis, the Mongolian wild ass in Kalamaili National Park was selected as the target species in this study. Potential habitat suitability was predicted using the BIOMOD2 ensemble modeling framework under current environmental conditions and future SSP126, SSP370, and SSP585 climate scenarios. Separately, current habitat-suitability predictions were spatially overlaid with soil-contamination classes derived from the Nemerow integrated pollution index to examine their spatial correspondence. Specifically, this study aimed to (1) identify the key environmental predictors associated with potential habitat suitability for the Mongolian wild ass; (2) assess changes in the extent and spatial distribution of suitable habitats under different future climate scenarios; and (3) quantify the spatial correspondence between current habitat suitability and soil heavy-metal contamination levels. By integrating these complementary assessments, this study provides a spatial basis for identifying conservation-priority habitats and areas requiring enhanced environmental monitoring in Kalamaili National Park.

## 2. Materials and Methods

### 2.1. Study Area

**Figure 1:**
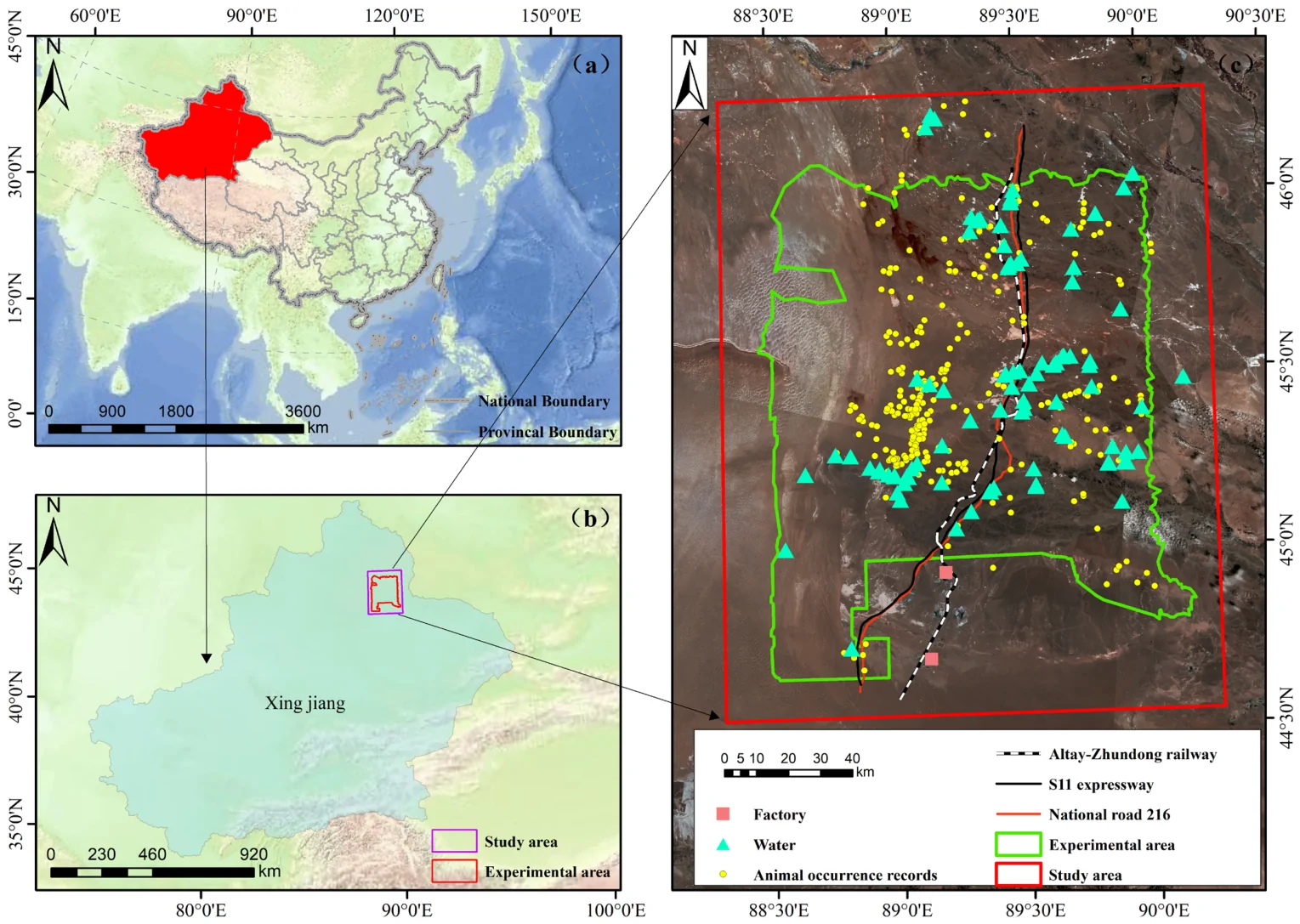
Schematic diagram of the study area. (**a**) Location of Xinjiang in China; (**b**) location of the study area within Xinjiang; (**c**) overview of the study area. (Map approval number: GS(2024)0650).

Xinjiang Kalamaili National Park is located on the eastern margin of the Junggar Basin, Xinjiang, northwestern China, between 88°26′–90°09′ E and 44°38′–46°03′ N. It spans parts of Changji Hui Autonomous Prefecture and Altay Prefecture, with a candidate-area extent of approximately 1.47 × 10^4^ km^2^ [24]. The region displays a typical temperate continental arid climate, marked by extended cold winters, short hot summers, and dry, warm spring and autumn seasons. The mean annual temperature is approximately 2.4 °C, with an annual precipitation of 159 mm and an annual evaporation of 2090 mm, resulting in an evaporation-to-precipitation ratio of approximately 13:1 [25]. Vegetation in Kalamaili National Park is dominated by desert vegetation, showing typical desert vegetation landscape characteristics of the Junggar Basin. This area also serves as an important habitat for desert ungulates and other protected wildlife in China, supporting 13 Class I and 36 Class II National Key Protected Wild Animal species [26]. Field surveys of the Mongolian wild ass were conducted both within Kalamaili National Park and in its surrounding areas, and some occurrence records were located outside the boundary of the national park candidate area. Therefore, restricting the modeling domain to the candidate area would have excluded these observed occurrence locations. To encompass both the candidate area and the recorded occurrence locations beyond its boundary, a rectangular modeling domain was manually delineated in ArcGIS 10.8, following the general approach of expanding the study extent to accommodate field-observed species distributions [27]. This domain was used consistently for environmental data processing, pseudo-absence sampling, and habitat-suitability projections. The final study area covered 2.9747 × 10^4^ km^2^ (Figure 1).

### 2.2. Data Sources and Processing

#### 2.2.1. Occurrence Data of the Mongolian Wild Ass

Occurrence records of the Mongolian wild ass were obtained from field surveys conducted from 3–10 September 2025. Surveys were carried out using the line-transect method, with a total of 21 transects established within Kalamaili National Park and its surrounding areas. Mongolian wild asses were detected through direct visual observations with binoculars, and the geographic coordinates of each independent observation were recorded using a GPS device. For group observations, one geographic location was recorded for each independent group encounter. A total of 324 occurrence records were initially obtained. After removing 10 records with duplicated geographic coordinates, 314 unique occurrence locations remained. Following a previously established spatial-filtering approach for Mongolian wild ass occurrence records [28], an 8 × 8 km grid was applied to reduce spatial clustering and redundancy. Within each occupied grid cell, only the occurrence record closest to the grid-cell centroid was retained. This procedure resulted in 109 spatially filtered occurrence records for subsequent modeling. Ten independent pseudo-absence datasets were generated by random sampling within the modeling domain [29]. Grid cells containing presence records were excluded from pseudo-absence sampling, and 1000 pseudo-absence points were generated for each dataset. The same 10 pseudo-absence datasets were subsequently used in the spatial block cross-validation procedure to account for the variation associated with pseudo-absence sampling.

#### 2.2.2. Collection of Environmental Variables

This study initially compiled 29 environmental predictors encompassing climate, topography, human activity, and habitat variables.

(1) Climatic variables: Current climatic variables were obtained from the WorldClim version 2.1 global climate database, representing the 1970–2000 baseline period and including 19 bioclimatic variables. Future climate projections were obtained from downscaled CMIP6 datasets for three Shared Socioeconomic Pathway scenarios, SSP126, SSP370, and SSP585, based on the BCC-CSM2-MR global climate model. Four future periods were considered: 2021–2040, 2041–2060, 2061–2080, and 2081–2100. Both current and future climatic layers were used at a spatial resolution of 30 arc-seconds;

(2) Topographic variables: The elevation dataset was obtained from the Geospatial Data Cloud platform of the Chinese Academy of Sciences. Based on this dataset, slope and aspect layers were derived using ArcGIS 10.8;

(3) Human activity variables: Road network information was extracted from the OpenStreetMap database, while factory locations were obtained from field surveys conducted in 2024. Distances to roads and factory locations were then calculated using ArcGIS 10.8. Human Footprint data were obtained from the Last of the Wild Project, Version 3 (LWP-3), provided by SEDAC. This dataset represents human pressure in 2009 at an original spatial resolution of approximately 1 km and integrates multiple anthropogenic pressures, including population density, land use, transportation, and infrastructure;

(4) Habitat variables: The vegetation type dataset was sourced from the 1:1,000,000-scale Chinese vegetation map issued by the National Tibetan Plateau Data Center. NPP data for 2025 were obtained from NASA Earthdata with a spatial resolution of 500 m. The NDVI dataset was sourced from MOD13A3. Based on the monthly NDVI raster layers for the 12 months of 2024, the maximum value was calculated to generate the annual NDVI data for that year. Water-source locations were obtained from field investigations conducted in Kalamaili National Park in 2025, and distances to water sources were then calculated using ArcGIS 10.8.

**Figure 2:**
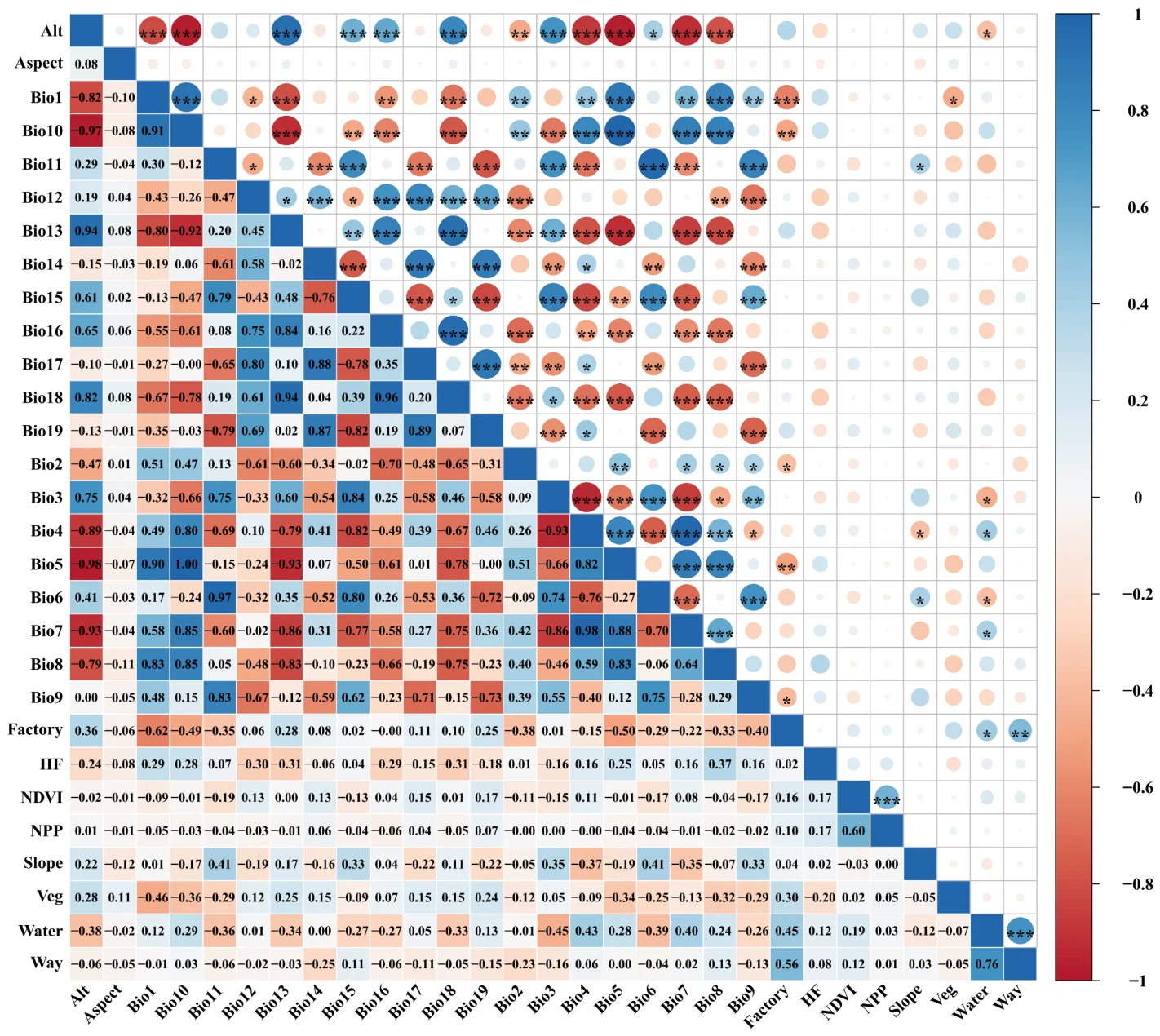
Correlation analysis of environmental variables. Asterisks indicate statistical significance (* *p* < 0.05, ** *p* < 0.01, and *** *p* < 0.001).

All environmental variables were clipped, projected, and resampled to a common spatial resolution prior to modeling. To minimize the influence of multicollinearity among predictors on model performance, Pearson correlation analysis was performed on the environmental raster layers using the ENMTools package in R version 4.2.3 (Figure 2), followed by variance inflation factor (VIF) analysis. Variables showing strong correlations (|r| ≥ 0.8) were first screened based on the Pearson correlation results. Additional redundant variables were then removed according to VIF values, with VIF ≤ 10 used as the retention criterion [30,31]. Following variable selection, 13 environmental predictors were retained for BIOMOD2 ensemble modeling, as shown in Table 1.

For future habitat suitability projections, all retained non-climatic predictors were held constant at their reference values, while only the retained climatic predictors were replaced with future climate projections under the SSP scenarios. Therefore, the future projections primarily reflect changes in habitat suitability associated with future climatic conditions under fixed non-climatic settings.

#### 2.2.3. Acquisition and Processing of Heavy-Metal Pollution Data

Soil sampling was conducted in July 2024 and September 2025. Soil sampling locations were randomly selected within the surveyed area. A total of 254 surface-soil samples (0–20 cm) were initially collected using a five-point sampling design within 5 m × 5 m quadrants, with five subsamples combined into one composite sample. After removing duplicated and closely located samples, 213 valid samples were retained. Samples were air-dried, ground, and sieved before analysis.

For Cu, Zn, Pb, Cr, and Cd determination, 0.30 g of soil was digested with 6 mL HCl and 2 mL HNO_3_ using a TANK ECO microwave digestion system (Sineo Microwave Chemistry Technology Co., Ltd., Shanghai, China) and analyzed using an Agilent 7900 ICP-MS (Agilent Technologies, Santa Clara, CA, USA). For As and Hg, 0.50 g of soil was digested using the same acid system, followed by pretreatment with a thiourea–ascorbic acid solution and measured using a PF52 atomic fluorescence spectrometer (Beijing Purkinje General Instrument Co., Ltd., Beijing, China).

Method detection limits for As, Cd, Cr, Cu, Hg, Pb, and Zn were 0.04, 0.01, 0.22, 3.79, 0.001, 0.17, and 1.01 mg·kg^−1^, respectively. Quality control included blank measurements, certified reference materials, and replicate analyses of approximately 10% of the samples. Reference materials were analyzed in triplicate, and measured values were within ±10% of the certified values.

### 2.3. Research Methods

#### 2.3.1. Model Construction and Evaluation

In this study, occurrence records of the Mongolian wild ass together with the corresponding environmental variables were used to develop habitat-suitability models within the BIOMOD2 framework (biomod2 package, version 4.2-6-2). Twelve modeling algorithms available in BIOMOD2 were considered, including ANN, CTA, FDA, MaxEnt, GAM, GBM, GLM, MARS, RF, RFd, SRE, and XGBOOST.

Model-specific parameters were configured using the predefined “bigboss” strategy in BIOMOD2. To minimize the potential for overestimating predictive performance due to spatial correlation between the calibration and validation data, model performance was evaluated using four-fold spatial block cross-validation [32,33,34]. Records of occurrences and pseudo-absences were divided into four geographically distinct blocks based on their spatial locations. In each iteration, three blocks were used for model calibration, while the remaining block was used for spatial validation. This process was repeated until each block had been used once as the validation dataset, and was applied across ten independently generated datasets representing pseudo-absences. Within each spatial cross-validation iteration, individual model runs with calibration TSS values of at least 0.5 were retained for EMca ensemble construction. A separate EMca ensemble was constructed for each pseudo-absence dataset and spatial validation fold. Predictive performance was evaluated using the area under the receiver operating characteristic curve (AUC) and the True Skill Statistic (TSS) [35,36]. For each spatial fold, the validation scores were first averaged across the ten pseudo-absence datasets. Overall predictive performance was then summarized as the mean ± standard deviation of the four fold-level means.

Following spatial cross-validation, the final models were fitted using all available occurrence records and each corresponding pseudo-absence dataset. The retained full-data models were combined into a final EMca ensemble, which was used to generate habitat-suitability projections under current and future climate scenarios.

Variable importance was assessed using the permutation-based procedure implemented in BIOMOD2, with three permutations performed for each environmental predictor. Importance scores for individual algorithms were averaged across the available full-data model runs and permutations, while those for the EMca ensemble were obtained separately from the final ensemble model. For comparison and visualization, the resulting importance scores were normalized within each model so that the relative importance of all predictors summed to 100%.

#### 2.3.2. Classification of Suitable Habitats

The continuous habitat-suitability outputs generated by the ensemble model were analyzed in ArcGIS 10.8. For the baseline classification, habitat suitability (H) was divided into four classes: unsuitable habitat (H ≤ 0.25), low-suitability habitat (0.25 < H ≤ 0.50), moderately suitable habitat (0.50 < H ≤ 0.75), and highly suitable habitat (H > 0.75) [37].

Changes in suitable habitat between the current and each future climate scenario were evaluated using the same H > 0.25 threshold. Grid cells that changed from unsuitable under current conditions to suitable under a future scenario were classified as expansion, whereas cells that changed from suitable to unsuitable were classified as contraction. Cells remaining suitable or unsuitable were classified as stable suitable or stable unsuitable, respectively.

To assess whether the estimated extent of suitable habitat and the estimated area of highly suitable habitat were sensitive to the predefined classification thresholds, two targeted sensitivity analyses were conducted. First, the threshold separating unsuitable from suitable habitat was varied from the baseline value of 0.25 to 0.20 and 0.30, and the total suitable habitat area was recalculated under the current and future climate scenarios. Second, the threshold defining highly suitable habitat was varied from the baseline value of 0.75 to 0.70 and 0.80, and the corresponding highly suitable habitat areas were recalculated. Threshold sensitivity was assessed because classification decisions can influence estimates of habitat extent and the spatial identification of conservation-priority areas [38]. The habitat-area estimates obtained under the alternative thresholds were compared with those based on the baseline thresholds to determine whether the direction of change across future periods and climate scenarios remained consistent (Appendix A, Table A1).

#### 2.3.3. Heavy-Metal Pollution Assessment and Spatial Interpolation

To characterize soil heavy-metal contamination, the single-factor pollution index and the Nemerow integrated pollution index were calculated using Xinjiang soil background values as reference values [39]. The single-factor pollution index was calculated as:

(1) $$P_{i}=\frac{C_{i}}{S_{i}}$$

where *P_i_* is the single-factor pollution index of metal *i*, *C_i_* is the concentration of metal *i*, and *S_i_* is the corresponding Xinjiang soil background value (Table A2). Based on the single-factor pollution index, soil-contamination levels were classified into four categories according to the criteria shown in Table 2.

The single-factor pollution indices and the numbers of soil samples within each pollution category for the seven heavy metals are summarized in Table A3.

The measured concentrations of Cr, Cu, Zn, Cd, Pb, Hg, and As at 213 sampling sites were interpolated using empirical Bayesian kriging (EBK) in ArcGIS 10.8. Model-specific transformations and semivariogram models are provided in Table A4, together with cross-validation statistics and prediction uncertainty. The EBK-predicted concentration surfaces were then converted to single-factor pollution-index rasters using the corresponding background values. For each raster cell, the maximum (*P_imax_*) and mean (*P_imean_*) values of the seven *P_i_* rasters were calculated, and the Nemerow integrated pollution index was obtained as:

(2) $$P_{N}=\sqrt{\frac{P_{\mathrm{imean}}^{2}+P_{\mathrm{imax}}^{2}}{2}}$$

The resulting *P_N_* values were classified according to the five levels shown in Table 3 [40].

For the subsequent spatial overlay analysis, the final Nemerow contamination raster was aligned with the habitat-suitability raster in terms of coordinate system, spatial extent, and cell resolution.

#### 2.3.4. Spatial Overlay of Current Habitat Suitability and Soil-Contamination Classes

Drawing on the concept of two-dimensional habitat assessment frameworks that combine habitat conditions with an independent risk or pressure dimension [41], habitat suitability and soil heavy-metal contamination were treated as two independent spatial dimensions. Habitat suitability (H) was derived from the BIOMOD2 ensemble predictions, whereas soil contamination was represented by the Nemerow integrated pollution index (*P_N_*). The Nemerow index was used to characterize relative spatial differences in soil heavy-metal contamination based on regional background values.

For the current spatial-overlay analysis, habitat suitability was divided into four classes according to the habitat suitability index (H): unsuitable (H ≤ 0.25), low suitability (0.25 < H ≤ 0.50), moderate suitability (0.50 < H ≤ 0.75), and high suitability (H > 0.75). Soil contamination retained the five Nemerow classes defined in Table 3: no pollution (*P_N_* ≤ 0.7), warning level (0.7 < *P_N_* ≤ 1.0), light pollution (1.0 < *P_N_* ≤ 2.0), moderate pollution (2.0 < *P_N_* ≤ 3.0), and heavy pollution (*P_N_* > 3.0).

The current habitat-suitability raster and the Nemerow contamination raster were aligned to a common spatial grid and spatially cross-tabulated in ArcGIS 10.8. The area corresponding to each combination of habitat-suitability class and soil-contamination class was then calculated.

The spatial-overlay analysis was restricted to current environmental conditions. Future habitat suitability was projected under the SSP126, SSP370, and SSP585 scenarios, whereas future soil-contamination patterns were not projected because reliable future heavy-metal contamination data were unavailable.

## 3. Results

### 3.1. Environmental Variable and Model Selection and Importance Assessment

**Figure 3:**
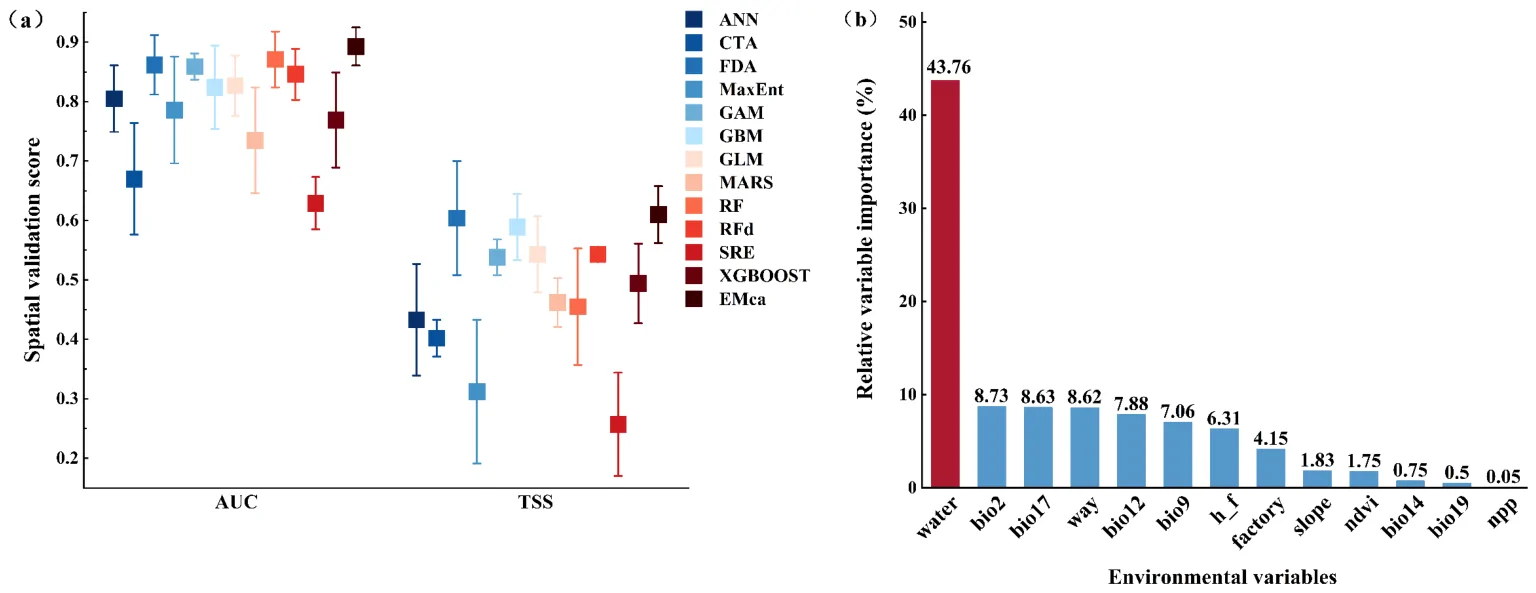
Spatial predictive performance and environmental variable importance. (**a**) Mean AUC and TSS values of individual models and the EMca ensemble under four-fold spatial block cross-validation, with error bars representing standard deviations. (**b**) Relative importance of the 13 environmental predictors in the final EMca ensemble model.

The twelve individual algorithms exhibited differences in predictive performance under four-fold spatial block cross-validation (Figure 3a). The EMca ensemble achieved an AUC of 0.893 ± 0.032 and a TSS of 0.610 ± 0.048, exhibiting higher mean predictive performance than all individual algorithms based on both evaluation metrics.

The variable-importance results showed that distance to water sources was the most influential predictor in the final EMca ensemble, accounting for 43.76% of the total normalized variable importance (Figure 3b). The next most important predictors were the mean diurnal range (Bio2, 8.73%), precipitation of the driest quarter (Bio17, 8.63%), distance to roads (Way, 8.62%), annual precipitation (Bio12, 7.88%), and mean temperature of the driest quarter (Bio9, 7.06%). These results highlighted the predominant importance of water-source conditions in predicting habitat suitability, while climatic variables and human-related factors were also important contributors to the modeled distribution of suitable habitats.

### 3.2. Spatial Patterns of Potential Suitable Habitats and Changes in Habitat Quality for the Mongolian Wild Ass Under Climate Change

**Figure 4:**
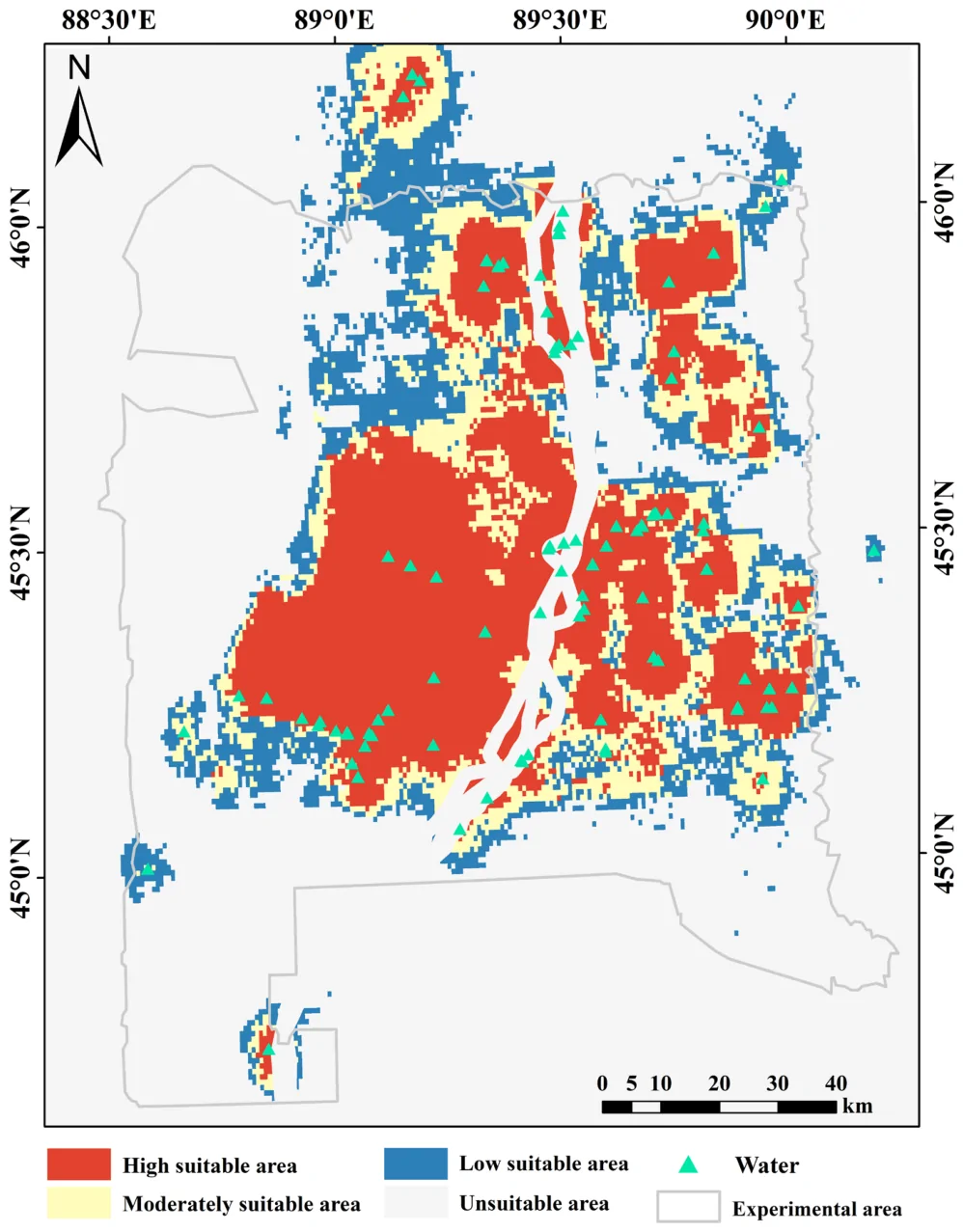
Distribution of suitable habitats for the Mongolian wild ass under current environmental conditions.

The predictions of the EMca ensemble model indicated that the potential suitable habitats for the Mongolian wild ass displayed distinct spatial clustering within the study area (Figure 4). Under current environmental conditions, the total suitable habitat area was 0.9256 × 10^4^ km^2^. Highly suitable, moderately suitable, and low-suitability habitats covered 0.4637 × 10^4^ km^2^, 0.2249 × 10^4^ km^2^, and 0.2370 × 10^4^ km^2^, respectively, accounting for 50.10%, 24.30%, and 25.61% of the total suitable habitat area.

In terms of spatial distribution, highly suitable habitat was predominantly located in the central and south-central regions of the study area, exhibiting a generally continuous patch distribution. Moderately suitable habitats were primarily situated adjacent to highly suitable habitats, functioning to some extent as transitional and buffer zones. The low-suitability habitat was predominantly located along the northern boundary and in certain southern regions of the study area, exhibiting a relatively dispersed spatial distribution.

**Figure 5:**
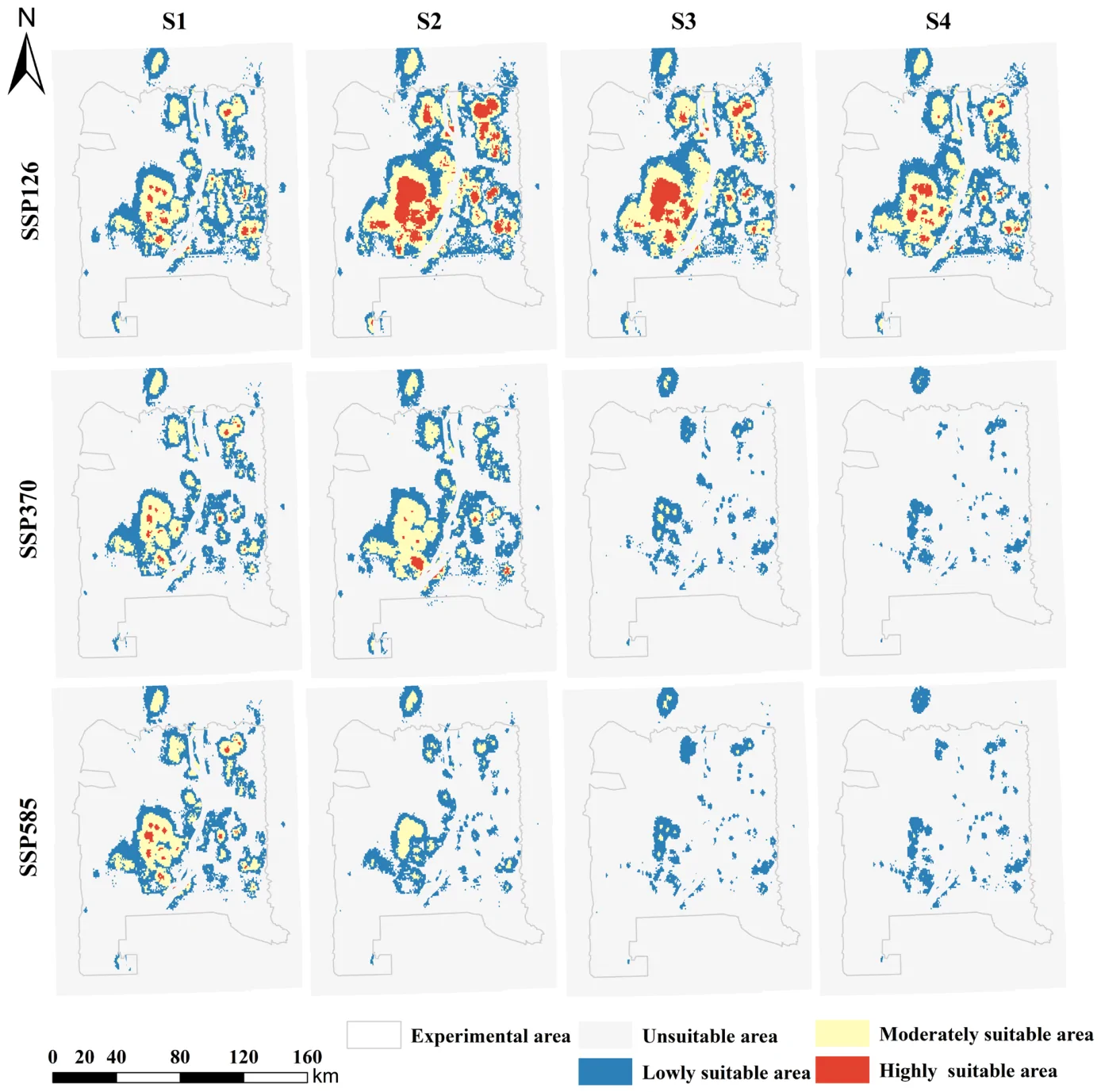
Distribution of suitable habitats for the Mongolian wild ass (S1: 2021–2040; S2: 2041–2060; S3: 2061–2080; S4: 2081–2100).

Across different future climate scenarios, both the extent and suitability-level structure of the Mongolian wild ass’s potential suitable habitats changed considerably (Figure 5, Table 4). Overall, the total suitable habitat area was lower than that under current conditions, although the magnitude of reduction varied among future periods and scenarios. Under SSP126, the total suitable habitat area was 0.5720 × 10^4^ km^2^ in 2021–2040, increased to 0.7713 × 10^4^ km^2^ in 2041–2060, and subsequently decreased to 0.7049 × 10^4^ km^2^ and 0.6420 × 10^4^ km^2^ in 2061–2080 and 2081–2100, respectively. Under SSP370, the total suitable habitat area changed from 0.4703 × 10^4^ km^2^ in 2021–2040 to 0.1143 × 10^4^ km^2^ in 2081–2100. A similar overall decline was observed under SSP585, with the total suitable habitat area decreasing from 0.4957 × 10^4^ km^2^ to 0.1371 × 10^4^ km^2^ over the same period. Overall, reductions in suitable habitat became more pronounced under SSP370 and SSP585 during the middle and late future periods.

In terms of changes among different suitability levels, highly suitable habitat showed the most pronounced decline under future climate scenarios (Table 4). The current area of highly suitable habitat was 0.4637 × 10^4^ km^2^, but decreased to 0–0.1180 × 10^4^ km^2^ across the future scenarios. The largest future area of highly suitable habitat occurred under SSP126 in 2041–2060 (0.1180 × 10^4^ km^2^). In contrast, no highly suitable habitat was predicted under SSP585 in 2041–2060, or under SSP370 and SSP585 during 2061–2080 and 2081–2100. Moderately suitable habitat also showed substantial variation among future periods and scenarios, ranging from 0.0015 × 10^4^ to 0.3033 × 10^4^ km^2^. Low-suitability habitat accounted for a relatively large proportion of the remaining suitable habitat in many future scenarios, with its area ranging from 0.1128 × 10^4^ to 0.3724 × 10^4^ km^2^. These results indicate that future habitat change was characterized not only by an overall reduction in suitable habitat area, but also by a marked decline in highly suitable habitat and a shift toward lower suitability levels.

Because the above changes in total suitable habitat and highly suitable habitat were derived from predefined classification thresholds, we further examined whether these area-based patterns were sensitive to threshold selection (Table A1). As expected, changing the suitable habitat threshold from H > 0.20 to H > 0.30 altered the absolute area estimates, with higher thresholds yielding smaller suitable habitat areas. However, the overall temporal and scenario-specific patterns remained similar: suitable habitat areas under future scenarios were consistently lower than those under current conditions, SSP126 generally retained larger suitable habitat areas, and the reductions were more pronounced under SSP370 and SSP585 during the middle and late future periods. A similar pattern was observed for highly suitable habitat. Although its estimated area decreased as the threshold increased from H > 0.70 to H > 0.80, highly suitable habitat declined markedly under future climate scenarios and became extremely limited or absent under SSP370 and SSP585 during the middle and late future periods. Overall, the main area-based trends identified using the baseline thresholds were broadly consistent under the alternative threshold settings.

From the perspective of spatial pattern changes, the current suitable habitats of the Mongolian wild ass were mainly distributed as relatively continuous patches in the central and south-central parts of the study area (Figure 4). Under future climate scenarios, these suitable habitat patches generally showed varying degrees of contraction and fragmentation, particularly under SSP370 and SSP585 in the middle and late future periods (Figure 5).

**Figure 6:**
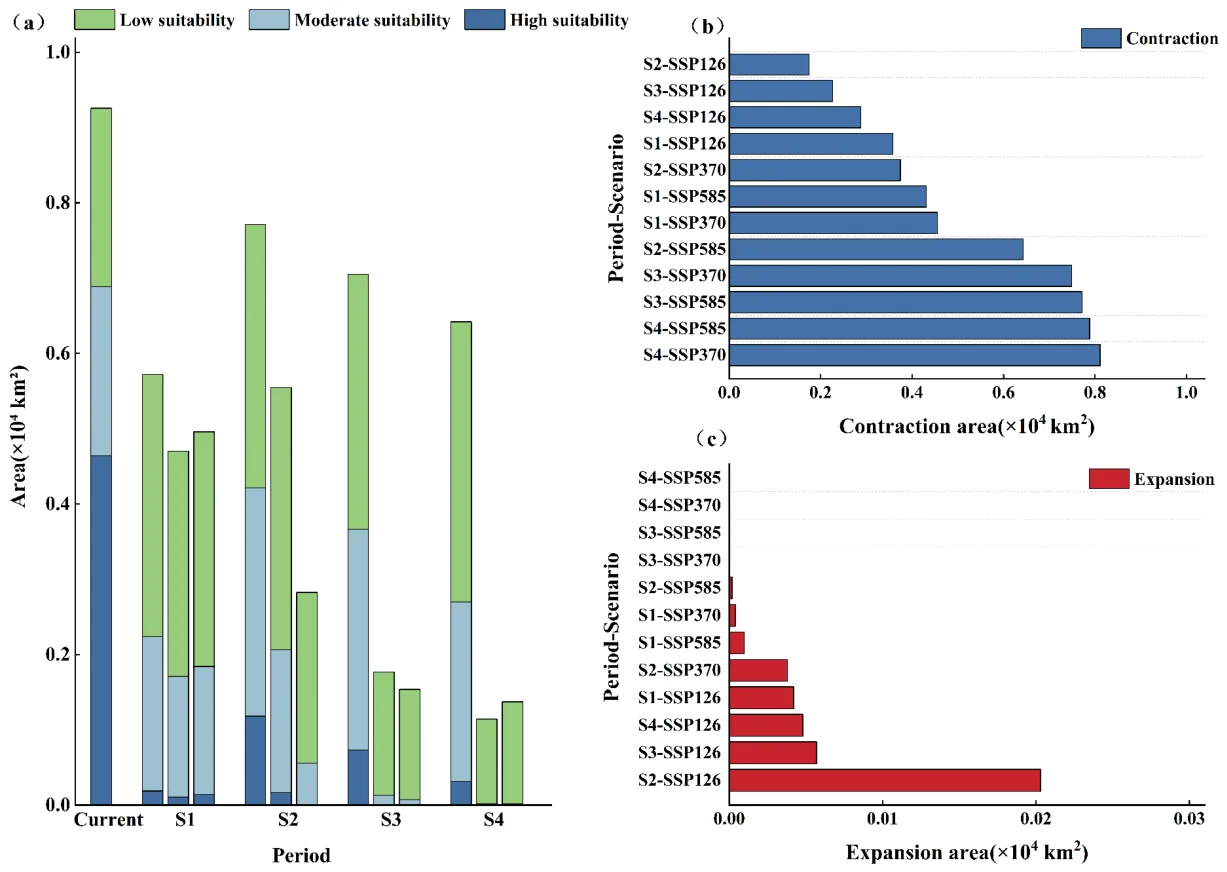
Changes in suitable habitat areas under current and future climate scenarios: (**a**) habitat suitability classes; (**b**) habitat contraction; and (**c**) habitat expansion. In (**a**), the three bars within each future period represent SSP126, SSP370, and SSP585 from left to right.

According to Figure 6 and Table 4, habitat contraction was consistently greater than habitat expansion across all future scenarios and periods. The expansion area ranged from 0 to 0.0203 × 10^4^ km^2^, whereas the contraction area ranged from 0.1745 × 10^4^ to 0.8113 × 10^4^ km^2^. The largest expansion occurred under SSP126 in 2041–2060, while the greatest contraction occurred under SSP370 in 2081–2100. During 2061–2080 and 2081–2100, no habitat expansion was detected under SSP370 or SSP585, whereas contraction remained substantial. These results indicate that future changes in suitable habitat were dominated by contraction, with the strongest losses occurring under the SSP370 and SSP585 scenarios during the middle and late future periods.

### 3.3. Soil Heavy-Metal Contamination and Its Spatial Correspondence with Habitat Suitability

#### 3.3.1. Spatial Patterns of Soil Heavy-Metal Concentrations and Contamination

Empirical Bayesian kriging (EBK) was used to generate continuous concentration surfaces for Cr, Cu, Zn, Cd, Pb, Hg, and As. Cross-validation showed generally small prediction biases, with the mean errors and mean standardized errors close to zero. RMSSE values ranged from 0.9615 to 1.0701, while RMSE and ASE were generally of similar magnitude for most metals. Prediction standard errors also varied among metals and across the interpolation domain (Table A4).

**Figure 7:**
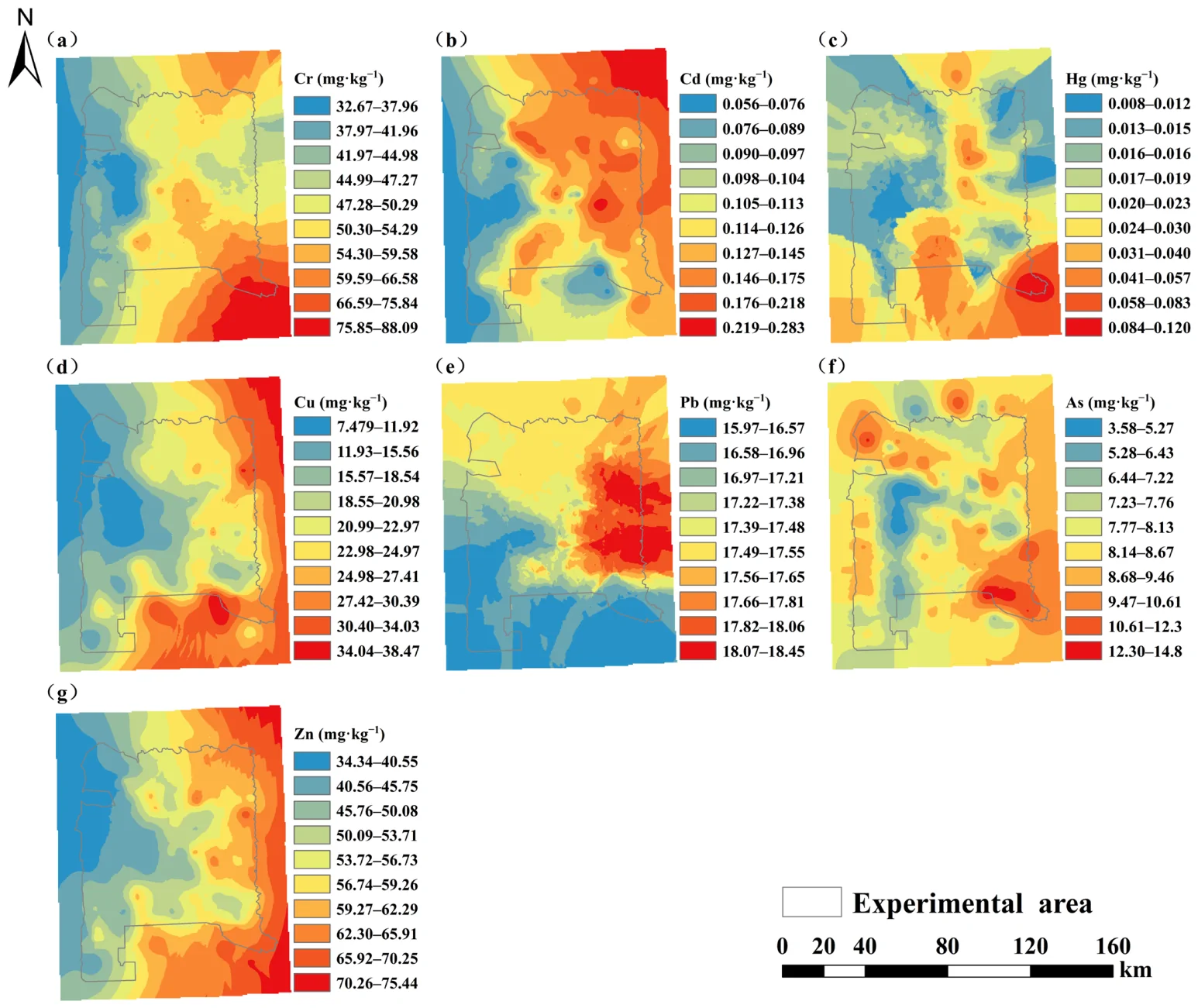
Spatial distributions of EBK-predicted concentrations of seven soil heavy metals in the study area: (**a**) Cr, (**b**) Cd, (**c**) Hg, (**d**) Cu, (**e**) Pb, (**f**) As, and (**g**) Zn.

The EBK-predicted concentration surfaces showed clear spatial heterogeneity among the seven heavy metals (Figure 7). The Cr concentrations were relatively elevated in the eastern and southeastern parts of the study area, whereas lower concentrations occurred mainly in the western sector. Cd showed relatively high concentrations in the northern and northeastern regions, together with several localized high-concentration patches in the central–eastern area. Hg exhibited pronounced local aggregation, with higher concentrations occurring mainly along the north-central to south–central portion of the study area and in the southeast. Cu concentrations were relatively high in the southern and southeastern regions and along parts of the eastern margin. Pb showed a distinct high-concentration cluster in the central–eastern part of the study area, while lower concentrations were mainly distributed in the southern and southwestern regions. As concentrations showed several localized high-concentration patches in the northwestern, north–central, and southeastern regions. Zn showed a generally increasing concentration pattern from west to east, with higher concentrations mainly occurring in the eastern and southeastern parts of the study area.

**Figure 8:**
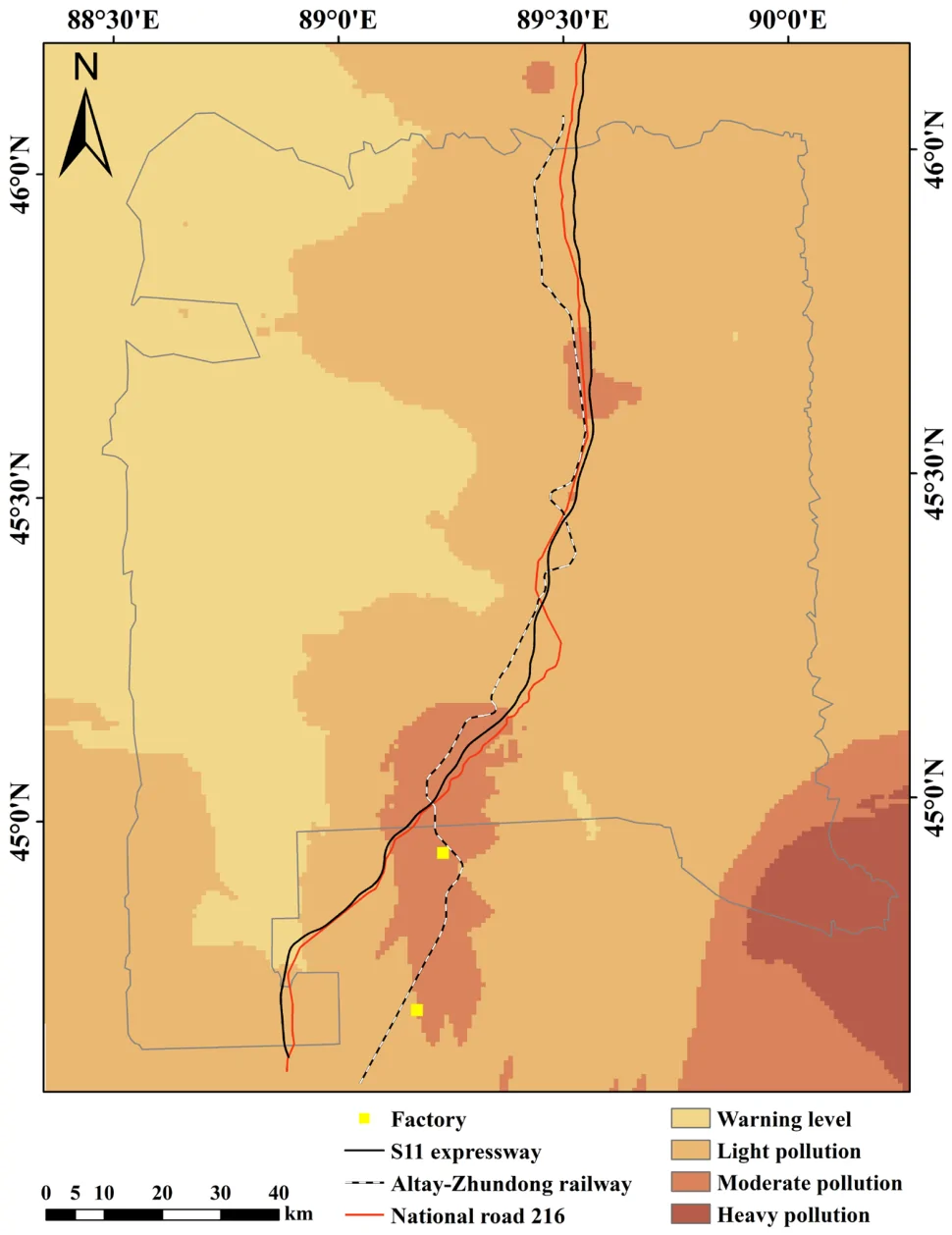
Spatial distribution of Nemerow integrated soil heavy-metal contamination classes in the study area.

The EBK-predicted concentration surfaces were subsequently converted to single-factor pollution-index rasters using the corresponding Xinjiang soil background values, and the Nemerow integrated pollution index was calculated from the seven *P_i_* rasters. No area within the study area fell within the no-pollution class (*P_N_* ≤ 0.7). Warning-level contamination accounted for 27.29% of the study area, whereas light pollution was dominant, covering 61.57%. Moderate and heavy pollution accounted for 7.11% and 4.02%, respectively (Figure 8). Spatially, higher Nemerow contamination classes were mainly concentrated in the south–central, southern, and southeastern parts of the study area, with some areas located close to major transportation corridors and industrial areas.

#### 3.3.2. Spatial Correspondence Between Current Habitat Suitability and Soil-Contamination Classes

**Figure 9:**
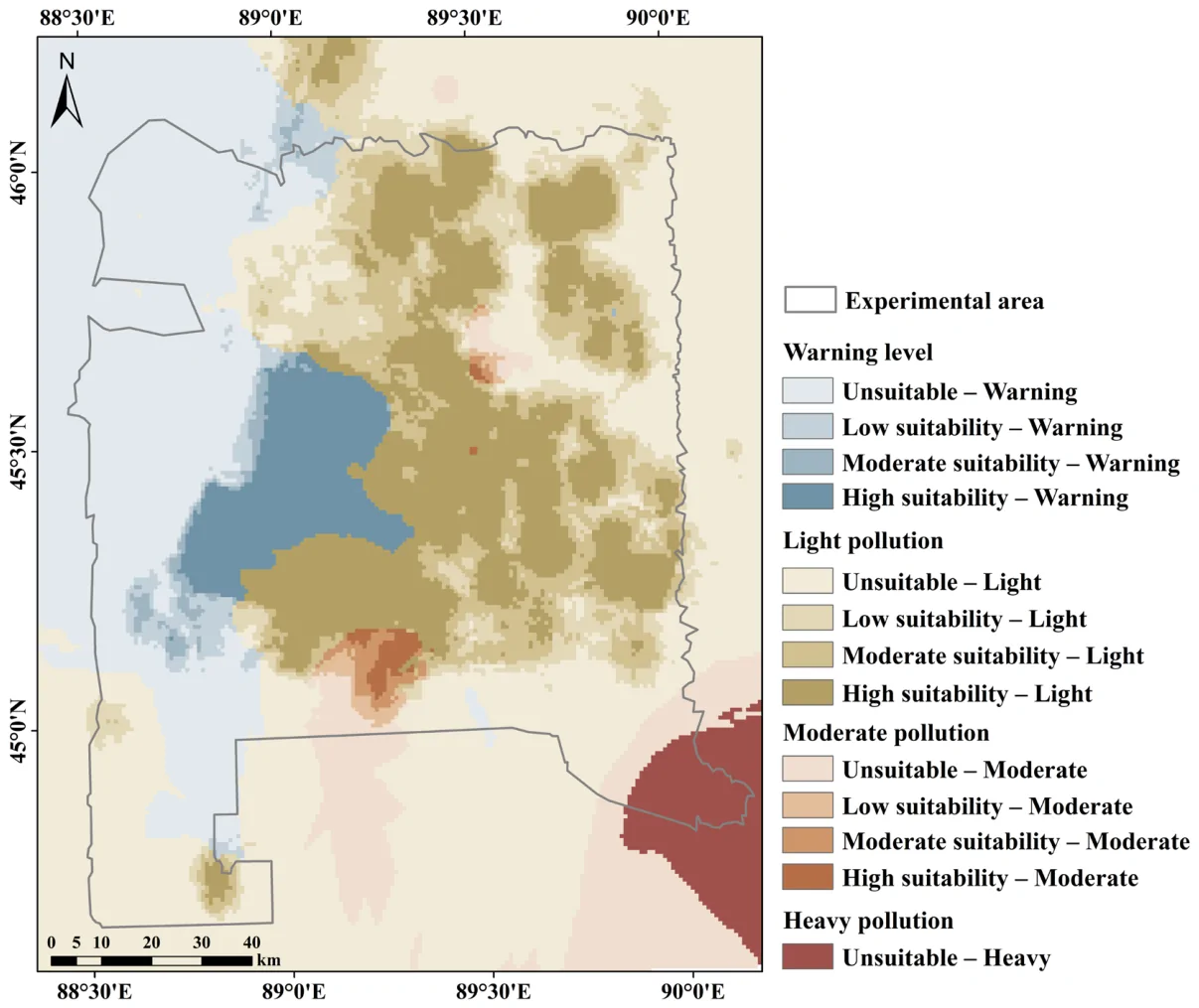
Spatial overlay of current habitat-suitability classes and Nemerow soil-contamination classes in the study area.

The spatial overlay of current habitat suitability and Nemerow soil-contamination classes showed clear differences in contamination conditions among habitat-suitability classes (Figure 9; Table 5). High-suitability habitat covered 0.4637 × 10^4^ km^2^. Of this area, 0.1128 × 10^4^ km^2^ (24.31%) overlapped with warning-level contamination, 0.3411 × 10^4^ km^2^ (73.56%) with light pollution, and 0.0098 × 10^4^ km^2^ (2.12%) with moderate pollution. No high-suitability habitat overlapped with the heavy-pollution class.

Moderate-suitability habitat covered 0.2249 × 10^4^ km^2^, of which 87.25% occurred within the light-pollution class. Relatively small proportions overlapped with warning-level contamination (7.89%) and moderate pollution (4.85%), while no overlap with heavy pollution was detected. Low-suitability habitat covered 0.2370 × 10^4^ km^2^ and showed a similar pattern, with 75.83% occurring within the light-pollution class, 20.16% within the warning-level class, and 4.01% within the moderate-pollution class. No low-suitability habitat overlapped with heavy pollution.

Spatially, high-suitability habitat overlapping warning-level contamination was mainly located in the central–western part of the study area, while most high-suitability habitat was associated with light pollution in the central region. Overlap between high suitability and moderate pollution was limited to a few small patches. Heavy-pollution areas were concentrated in the southeast and occurred only within unsuitable habitat (Figure 9).

Overall, the spatial correspondence analysis showed that high habitat suitability did not necessarily coincide with the lowest levels of soil contamination. Although most of the highly suitable habitat occurred within warning-level or light-pollution areas, a small proportion also overlapped with moderate pollution. These results indicate that habitat suitability and soil-contamination conditions provide complementary spatial information and that considering both dimensions can help distinguish areas with high habitat suitability from locations where contamination monitoring may require greater attention.

## 4. Discussion

### 4.1. Effects of Water-Source Conditions on Habitat Suitability for the Mongolian Wild Ass in Arid Regions

In arid regions, water sources are pivotal factors affecting the distribution of ungulates [13,42]. Kalamaili National Park is characterized by low annual precipitation and high evaporation [25], while water sources are unevenly distributed across the landscape [27]. Water sources not only provide drinking opportunities for the Mongolian wild ass but also influence its habitat use and movements in arid environments [22,43]. The findings of this study indicated that, among all 13 environmental predictors, distance to water sources was the most important environmental predictor, with a relative importance of 43.76% in the final EMca ensemble model (Figure 3b), highlighting the important role of water-source conditions in predicting habitat suitability for the Mongolian wild ass.

The central and south–central parts of the study area are characterized by abundant water sources, relatively flat terrain, and high vegetation cover, which together provide ideal environmental conditions for foraging, drinking, and movement of the Mongolian wild ass [44]. This study identified that areas of elevated habitat suitability for the Mongolian wild ass were predominantly distributed across the central and south–central portions of the study area (Figure 4). This outcome aligns with prior research indicating that appropriate habitats for the Mongolian wild ass in Kalamaili National Park are predominantly located in the central region of the protected area [28,45]. However, this concentrated distribution also indicates that the Mongolian wild ass has a high dependence on key water sources and limited forage areas. Once these resource patches are affected by climate change, human disturbance, or pollution risk, habitat stability may decline. In other words, the loss or degradation of key water sources may have an impact on habitat suitability, particularly in areas where water sources are scarce. Therefore, conservation of large herbivores in arid regions should not focus only on the total area of suitable habitats but should also pay particular attention to the ecological quality and pollution status of key water sources [22].

### 4.2. Projected Changes in Habitat Suitability Under Future Climate Scenarios

The model projections indicated substantial changes in the extent and suitability-class structure of potential habitat for the Mongolian wild ass under future climate scenarios. Highly suitable habitat showed the most pronounced decline, decreasing from 0.4637 × 10^4^ km^2^ under current conditions to 0–0.1180 × 10^4^ km^2^ across future scenarios. Total suitable habitat was also lower than the current value under all future scenarios, with particularly strong reductions under SSP370 and SSP585 during the middle and late future periods. Although low-suitability habitat accounted for a relatively large proportion of the remaining suitable habitat in many future scenarios, its absolute area did not increase consistently across all periods and scenarios. These projected changes suggest that future climatic conditions may be associated with not only an overall contraction of suitable habitat but also a restructuring of habitat suitability, characterized by a marked loss of highly suitable habitat. Previous studies have similarly projected reductions and increased vulnerability of Mongolian wild ass habitats under future climate scenarios [23,29]. Studies considering interactions between climate and land-use or landscape dynamics have further shown that multiple environmental changes can reshape habitat distribution and intensify habitat loss [14,46].

The pronounced decline in highly suitable habitat and the restructuring of habitat-suitability patterns may be closely related to the resource-constrained nature of arid ecosystems. Changes in temperature and precipitation may influence water availability and forage resources in arid ecosystems, potentially affecting habitat conditions for the Mongolian wild ass [8,10,47]. However, in the present study, non-climatic predictors, including distance to water sources, vegetation indices, and human-activity variables, were held constant in future projections. Therefore, the projected changes primarily reflect responses to future climatic conditions under fixed non-climatic settings, rather than the combined effects of future changes in water availability, vegetation, and human activities.

Although the Mongolian wild ass is highly mobile, its habitat use in arid desert regions remains strongly associated with the spatial distribution of water and forage resources [22,44,48]. Under future climate scenarios, the predicted reduction in highly suitable habitat and changes in its spatial distribution suggest a potential decline in the extent and continuity of core habitat areas, particularly under SSP370 and SSP585. These findings highlight the importance of protecting key water sources, maintaining core habitats, and preserving habitat connectivity in long-term conservation planning.

### 4.3. Implications of Soil Heavy-Metal Contamination for Habitat Suitability Assessment

Traditional species distribution models primarily characterize potential habitat suitability based on environmental predictors such as climate, topography, water availability, vegetation, and human disturbance, but they do not directly describe spatial variation in soil-contamination conditions. By overlaying current habitat suitability with Nemerow soil-contamination classes, this study showed that high-suitability habitat occurred across different contamination levels. Most high-suitability habitat overlapped with the light-pollution class (73.56%), followed by warning-level contamination (24.31%), while 2.12% overlapped with moderate pollution. No high-suitability habitat overlapped with the heavy-pollution class. These results indicate that high habitat suitability does not necessarily correspond to the lowest soil-contamination level and that habitat suitability alone cannot fully represent spatial variation in contamination conditions.

This spatial overlap is environmentally relevant because heavy metals such as Cd, Hg, and Cr can persist in soils and may enter the terrestrial food chain through forage, water, and soil or dust ingestion [5,17]. Heavy-metal contamination has also been reported in plant samples from Kalamaili National Park [15], suggesting that metals present in the soil environment may enter forage resources and represent potential exposure pathways for the Mongolian wild ass. A study of domestic horses exposed near non-ferrous metal smelters reported estimated daily Pb and Cd intakes of 6.0 and 1.1 mg kg^−1^ body weight day^−1^, respectively, together with elevated tissue metal concentrations and hematological abnormalities [49]. Therefore, areas where suitable habitats overlap with relatively elevated soil-contamination levels should be considered priorities for further investigation of potential heavy-metal exposure. These overlap areas warrant greater attention through targeted monitoring of soil, vegetation, and water quality.

### 4.4. Implications for the Management of Large Herbivores in Arid Protected Areas

Previous habitat-based conservation frameworks have emphasized the importance of considering habitat conditions together with independent environmental pressures when identifying conservation priorities [41]. In this study, habitat-suitability classes were spatially overlaid with Nemerow soil-contamination classes to quantify the area and distribution of their different combinations. The results showed that highly suitable habitats occurred under different soil-contamination conditions, suggesting that habitat suitability and soil contamination should be considered together when developing conservation and environmental-monitoring strategies.

From a management perspective, high-suitability habitats should remain a priority for conserving key water sources, forage resources, habitat continuity, and movement opportunities for the Mongolian wild ass [14,22]. At the same time, suitable habitats overlapping with relatively higher soil-contamination levels should receive greater attention through monitoring of soils, forage plants, and water sources, particularly in the south–central, southern, and southeastern parts of the study area, where higher Nemerow contamination levels were more concentrated. In addition, future monitoring could incorporate animal-based measurements, as demonstrated in previous wildlife biomonitoring studies [50,51]. Integrating habitat-suitability predictions with soil-contamination information can therefore help distinguish areas requiring habitat protection from those where additional environmental monitoring and pollution management may be needed.

Moreover, this study has several limitations. First, future spatial changes in soil heavy-metal contamination remain uncertain. Because reliable future heavy-metal contamination data were unavailable, the spatial overlay between habitat suitability and soil contamination was restricted to current environmental conditions. Future analyses therefore focused only on climate-driven changes in habitat suitability. In addition, the baseline climatic data and field observations were derived from different time periods. Therefore, the spatial overlay of habitat suitability and soil heavy-metal contamination should be interpreted as an assessment of their potential spatial correspondence rather than a direct representation of habitat conditions in a specific year. Second, non-climatic predictors, including slope, distance to water, NDVI, NPP, and human-activity variables, were kept unchanged in the future habitat-suitability projections. Therefore, the projected changes primarily reflect responses to future climatic conditions and do not account for potential future changes in water availability, vegetation productivity, or human disturbance. Third, although this study identified potential changes in habitat suitability and the spatial correspondence between suitable habitats and soil heavy-metal contamination, it did not directly assess heavy-metal exposure, physiological responses, or population-level effects in the Mongolian wild ass. Furthermore, the soil-contamination assessment was primarily based on soil samples and was not validated using heavy-metal measurements from water sources, forage plants, or fecal samples. Therefore, the findings should not be interpreted as evidence of direct biological impacts on the species, and further biological monitoring is needed to evaluate potential exposure pathways and health effects. Fourth, the occurrence records used in this study were collected during a limited survey period and may not fully represent seasonal variation in habitat use. Future studies combining multi-season field surveys with long-term GPS tracking would provide a more complete understanding of seasonal movements and habitat use. Fifth, although occurrence records were spatially filtered using an 8 × 8 km grid and model performance was evaluated using four-fold spatial block cross-validation with spatially separated validation folds, the evaluation results may still be influenced by the spatial configuration of the validation blocks and the sampling of pseudo-absences. Future studies incorporating independent occurrence datasets and additional spatial validation strategies could provide a more comprehensive assessment of model transferability and predictive robustness.

## 5. Conclusions

The present research employed a BIOMOD2-based ensemble modeling framework to project potential suitable habitats for the Mongolian wild ass under current environmental conditions and future climate scenarios. The spatial distribution of current soil heavy-metal contamination was further overlaid with current habitat suitability to examine their spatial correspondence. The primary conclusions are as follows:

(1) Water limitation was a key factor affecting the suitability of Mongolian wild ass habitats in arid regions. Distance to water sources had the highest relative importance (43.76%), highlighting the important role of water availability in shaping the predicted distribution of suitable habitats;

(2) Future climate change was projected to reduce the extent and quality of suitable habitats for the Mongolian wild ass. Highly suitable habitat decreased from 0.4637 × 10^4^ km^2^ under current conditions to 0–0.1180 × 10^4^ km^2^ across future climate scenarios. The total suitable habitat area also declined under all future scenarios, with the most pronounced reductions occurring under SSP370 and SSP585 during the middle and late future periods;

(3) The spatial overlay of current habitat suitability and soil-contamination classes showed that high-suitability habitats occurred under different contamination conditions. Of the current high-suitability habitat, 73.56% overlapped with light pollution, 24.31% with warning-level contamination, and 2.12% with moderate pollution, while no overlap with heavy pollution was observed. These results indicate that habitat suitability alone does not fully reflect spatial differences in soil-contamination conditions;

(4) Conservation management should prioritize high-suitability habitats and key water-source areas, while targeted monitoring of soils, forage plants, and water sources should be strengthened where suitable habitats overlap with relatively higher soil-contamination levels, especially in the south–central, southern, and southeastern parts of the study area.

## Figures and Tables

**Table 1:** Codes and descriptions of environmental variables.

Type	Environment Variable Description (Unit)	Code
Climate variables	Mean diurnal range	Bio 2
	Mean temperature of driest quarter	Bio 9
	Annual precipitation	Bio12
	Precipitation of driest month	Bio 14
	Precipitation of driest quarter	Bio 17
	Precipitation of coldest quarter	Bio 19
Topographic variables	Slope	Slope
Human activity variables	Distance to road	Way
	Distance to factory area	Factory
	Human Footprint	Human_Footprint
Habitat variables	Net primary productivity	NPP
	Normalized difference vegetation index	NDVI
	Distance to water source	Water

**Table 2:** Classification of soil-contamination levels based on the single-factor pollution index.

Pollution Index (*P_i_*)	Pollution Level
*P_i_* < 1	No pollution
1 ≤ *P_i_* ≤ 3	Light pollution
3 < *P_i_* ≤ 6	Moderate pollution
*P_i_* > 6	Heavy pollution

**Table 3:** Soil pollution assessment levels based on the Nemerow index.

Nemerow Comprehensive Pollution Index	Pollution Level	Assessment Result
*P_N_* ≤ 0.7	I	No pollution
0.7 < *P_N_* ≤ 1.0	II	Not yet polluted (Warning line)
1.0 < *P_N_* ≤ 2.0	III	Light pollution
2.0 < *P_N_* ≤ 3.0	IV	Moderate pollution
*P_N_* > 3.0	V	Heavy pollution

**Table 4:** Changes in the spatial distribution pattern of suitable habitats for the Mongolian wild ass under different climate scenarios.

Period	Scenario	High Suitability Habitat Area	Moderate Suitability Habitat Area	Low Suitability Habitat Area	Total Suitable Habitat Area	Expansion Area	Contraction Area
Current	-	0.4637	0.2249	0.2370	0.9256	—	—
2021—2040	SSP126	0.0187	0.2050	0.3482	0.5720	0.0042	0.3578
	SSP370	0.0107	0.1601	0.2994	0.4703	0.0004	0.4557
	SSP585	0.0139	0.1702	0.3117	0.4957	0.0010	0.4309
2041—2060	SSP126	0.1180	0.3033	0.3500	0.7713	0.0203	0.1745
	SSP370	0.0166	0.1899	0.3482	0.5547	0.0038	0.3747
	SSP585	0	0.0556	0.2269	0.2825	0.0002	0.6433
2061—2080	SSP126	0.0731	0.2931	0.3388	0.7049	0.0057	0.2263
	SSP370	0	0.0130	0.1637	0.1766	0	0.7489
	SSP585	0	0.0070	0.1467	0.1536	0	0.7719
2081—2100	SSP126	0.0313	0.2383	0.3724	0.6420	0.0048	0.2883
	SSP370	0	0.0015	0.1128	0.1143	0	0.8113
	SSP585	0	0.0017	0.1354	0.1371	0	0.7885

Notes: All data above are expressed in units of ×10^4^ km^2^.

**Table 5:** Spatial correspondence between current habitat suitability and Nemerow soil-contamination levels.

Habitat Suitability Class	Nemerow Soil-Contamination Level				Total
	Warning Level	Light Pollution	Moderate Pollution	Heavy Pollution	
Unsuitable	0.6335	1.1146	0.1813	0.1197	2.0491
Low suitability	0.0478	0.1797	0.0095	0	0.2370
Moderate suitability	0.0178	0.1962	0.0109	0	0.2249
High suitability	0.1128	0.3411	0.0098	0	0.4637
Total	0.8119	1.8316	0.2115	0.1197	2.9747

Note: Areas are expressed in units of ×10^4^ km^2^. Totals were calculated from unrounded values.

## Data Availability

The data presented in this study is available on request from the corresponding author. The data is not publicly available due to the ongoing nature of the research.

## Abbreviations

The following abbreviations are used in this manuscript:

Cr	Chromium
Cu	Copper
Zn	Zinc
Cd	Cadmium
Pb	Lead
Hg	Mercury
As	Arsenic

## Appendix A

**Table A1:** (a) Sensitivity of total suitable habitat area to alternative suitable habitat thresholds. (b) Sensitivity of highly suitable habitat area to alternative high-suitability thresholds.

**(a)**				
**Period**	**Scenario**	**Suitable Habitat Threshold**		
		**Less Restrictive (H > 0.20)**	**Baseline (H > 0.25)**	**More Restrictive (H > 0.30)**
Current	—	0.9888	0.9256	0.8696
2021–2040	SSP126	0.6828	0.5720	0.4881
	SSP370	0.5686	0.4703	0.3933
	SSP585	0.6130	0.4957	0.4056
2041–2060	SSP126	0.8874	0.7713	0.6793
	SSP370	0.6773	0.5547	0.4531
	SSP585	0.3867	0.2825	0.2109
2061–2080	SSP126	0.8193	0.7049	0.6167
	SSP370	0.2596	0.1766	0.1149
	SSP585	0.2272	0.1536	0.0989
2081–2100	SSP126	0.7702	0.6420	0.5347
	SSP370	0.1862	0.1143	0.0628
	SSP585	0.2208	0.1371	0.0754
**(b)**				
**Period**	**Scenario**	**Highly suitable habitat threshold**		
		**Less restrictive (H > 0.70)**	**Less restrictive (H > 0.70)**	**Less restrictive (H > 0.70)**
Current	—	0.5152	0.4637	0.4025
2021–2040	SSP126	0.0436	0.0187	0.0047
	SSP370	0.0279	0.0107	0.0016
	SSP585	0.0392	0.0139	0.0027
2041–2060	SSP126	0.1689	0.1180	0.0700
	SSP370	0.0347	0.0166	0.0082
	SSP585	0.0014	0	0
2061–2080	SSP126	0.1154	0.0731	0.0383
	SSP370	0.0001	0	0
	SSP585	0	0	0
2081–2100	SSP126	0.0662	0.0313	0.0077
	SSP370	0	0	0
	SSP585	0	0	0

(a) Note: H represents the continuous habitat-suitability value predicted by the ensemble model. Grid cells with H greater than the selected threshold were classified as suitable habitat. The baseline suitable/unsuitable threshold was H = 0.25, while H = 0.20 and H = 0.30 were used to evaluate threshold sensitivity. Areas are expressed in units of ×10^4^ km^2^. (b) Note: H represents the continuous habitat-suitability value predicted by the ensemble model. Grid cells with H greater than the selected high-suitability threshold were classified as highly suitable habitat. The baseline threshold was H = 0.75, while H = 0.70 and H = 0.80 were used to evaluate threshold sensitivity. Areas are expressed in units of ×10^4^ km^2^.

**Table A2:** Statistical characteristics of soil heavy-metal concentrations in the study area.

	Cr	Cu	Zn	Cd	Pb	Hg	As
Minimum (mg·kg^−1^)	16.30	5.10	24.71	0.03	9.15	0.003	1.73
Maximum (mg·kg^−1^)	173.83	62.92	115.21	0.34	25.71	0.223	21.40
Mean (mg·kg^−1^)	50.65	20.48	54.23	0.14	17.25	0.023	8.51
Standard deviation (mg·kg^−1^)	17.70	8.18	15.03	0.06	2.07	0.025	2.73
Xinjiang soil background value (mg·kg^−1^)	49.30	26.70	68.80	0.12	19.40	0.017	11.20

**Table A3:** Single-factor pollution index values and pollution-level classification of soil heavy metals in the study area.

	Cr	Cu	Zn	Cd	Pb	Hg	As
Maximum	3.53	2.36	1.67	2.85	1.33	13.12	1.91
Minimum	0.33	0.19	0.36	0.25	0.47	0.18	0.15
Mean	1.03	0.77	0.79	1.13	0.89	1.38	0.76
Number of unpolluted samples	112	170	183	87	190	103	194
Number of lightly polluted samples	100	43	30	126	23	94	19
Number of moderately polluted samples	1	0	0	0	0	12	0
Number of heavily polluted samples	0	0	0	0	0	4	0

**Table A4:** (a) EBK model settings for soil heavy-metal concentrations. (b) Cross-validation statistics and prediction uncertainty of the EBK models.

**(a)**							
		**Transformation**			**Semivariogram Model**		
Cr		Log-empirical			Exponential Detrended		
Cu		Empirical			K-Bessel Detrended		
Zn		Empirical			Exponential Detrended		
Cd		Log-empirical			Exponential Detrended		
Pb		Log-empirical			Exponential		
Hg		None			Power		
As		Empirical			Exponential		
**(b)**							
	**ME**	**RMSE**	**Mean Std. Error**	**RMSSE**	**ASE**	**Mean PSE**	**PSE range**
Cr	0.0909	16.5323	0.0069	1.0701	14.5919	7.5036	3.6044–20.7828
Cu	−0.0793	6.5017	−0.0232	1.0614	6.2785	4.7937	1.2524–13.9832
Zn	−0.0733	13.3964	−0.0152	1.0511	13.3684	7.9145	3.5313–16.2999
Cd	0.0002	0.0456	−0.0007	0.9615	0.0471	0.0350	0.0132–0.0962
Pb	−0.0280	1.9985	−0.0187	0.9865	1.9653	0.6057	0.2242–1.3891
Hg	−0.000021	0.0238	0.0005	0.9996	0.0220	0.0157	0.0088–0.0367
As	−0.0034	2.5475	−0.0066	1.0038	2.4679	1.8426	0.9251–3.4230

(b) Notes: **ME**, mean error; **RMSE**, root mean square error; **RMSSE**, root mean square standardized error; **ASE**, average standard error; **PSE**, prediction standard error. ME, RMSE, ASE, and PSE are expressed in mg·kg^−1^; standardized errors and RMSSE are dimensionless.
